# Results of the Phase 1 Open-Label Safety Study of Umbilical Cord Lining Mesenchymal Stromal/Stem Cells (Corlicyte^®^) to Heal Chronic Diabetic Foot Ulcers

**DOI:** 10.3390/biomedicines12061375

**Published:** 2024-06-20

**Authors:** Cecilia C. Low Wang, Tae Chong, Garrett Moore, Benjamin Echalier, Nicola Haakonsen, James E. Carter, David Mathes, Judith Hsia, Toan Thang Phan, Ivor J. Lim, Brian M. Freed

**Affiliations:** 1 Division of Endocrinology, Metabolism and Diabetes, Department of Medicine, School of Medicine, University of Colorado Anschutz Medical Campus, Aurora, CO 80045, USA; 2 Division of Plastic and Reconstructive Surgery, Department of Surgery, School of Medicine, Virginia Commonwealth University, Richmond, VA 23298, USA; tae.chong@vcuhealth.org; 3 Department of Orthopedics, School of Medicine, University of Colorado Anschutz Medical Campus, Aurora, CO 80045, USA; garrett.moore@cuanschutz.edu; 4 University of Colorado Anschutz Medical Campus, Aurora, CO 80045, USA; 5 Department of Cardiovascular Medicine, Miller Family Heart, Vascular, and Thoracic Institute, Cleveland Clinic, Cleveland, OH 44195, USA; carterj25@ccf.org; 6 Division of Plastic and Reconstructive Surgery, Department of Surgery, School of Medicine, University of Colorado Anschutz Medical Campus, Aurora, CO 80045, USA; david.mathes@cuanschutz.edu; 7 Division of Cardiology, Department of Medicine, School of Medicine, University of Colorado Anschutz Medical Campus, Aurora, CO 80045, USA; judith.hsia@cpcmed.org; 8 CPC Clinical Research, Aurora, CO 80045, USA; 9 Cell Research Corporation Pte Ltd., Singapore 048943, Singapore; ttphan@cellresearchcorp.com (T.T.P.); ivorlim@cellresearchcorp.com (I.J.L.); 10 Division of Allergy and Clinical Immunology, Department of Medicine, School of Medicine, University of Colorado Anschutz Medical Campus, Aurora, CO 80045, USA; brian.freed@cuanschutz.edu

**Keywords:** diabetic foot ulcer, diabetes, mesenchymal stem cell, safety, stem cell

## Abstract

Background: Mesenchymal stromal/stem cells (MSCs) play a critical role in wound healing. Corlicyte^®^ is an MSC product derived from allogeneic umbilical cord tissue donated under an institutional review board-approved protocol and processed in accordance with section 501(a)(2)(B) of the Federal Food, Drug, and Cosmetic Act. This open-label phase 1 trial was performed under a United States Food and Drug Administration Investigational New Drug Application to establish the safety and tolerability of Corlicyte^®^ in patients with diabetes and chronic diabetic foot ulcer (DFU). Methods: Escalating doses were applied topically twice a week for up to 8 weeks after ulcer debridement, wound photography, and measurement. Subjects were followed for 4 weeks after the treatment phase. Adverse events were assessed at every visit. Results: Nine subjects in 2 dosing cohorts completed the trial. No subjects experienced a serious adverse reaction to Corlicyte^®^ or the development of anti-human leukocyte antigen (HLA) antibodies. Sixty percentage of subjects in the lower dose cohort experienced ulcer closure by Day 70 of follow-up, while the mean ulcer size was reduced by 54–67% in the other subjects. Conclusions: Topical administration of Corlicyte^®^, a novel biologic therapy consisting of allogeneic umbilical cord lining MSCs, appeared safe and tolerable and resulted in a significant decrease in ulcer area, demonstrating its potential as a therapy for healing of chronic DFU.

## 1. Introduction

Up to 35% of individuals with diabetes develop diabetic foot ulcer (DFU) [1], yet less than half of DFU heal with standard of care [2]. Diabetes-related lower extremity complications negatively impact function and quality of life, and cost almost $80 billion in the United States in 2015 [3]. Numerous studies clearly demonstrate that mesenchymal stromal/stem cells (MSCs) play a critical role in wound healing [4,5,6,7,8,9,10,11,12]. Pre-clinical studies show that topical application of MSCs has a positive effect on ulcer healing in diabetes [13]. Few clinical studies have examined the use of topical application of MSCs in chronic DFU. The objective of the phase 1 trial of Corlicyte^®^ was to establish the safety and tolerability of Corlicyte^®^ MSCs for the treatment of patients with chronic DFU (ClinicalTrials.gov Identifier: NCT04104451).

This phase 1 trial demonstrated that topical administration of Corlicyte^®^ MSCs appeared to be safe and tolerable in patients with diabetes and chronic DFU. There was a significant decrease in ulcer area and warrants further study as a potential therapy for healing chronic DFU.

## 2. Materials and Methods

This was a single-center, single-arm, open-label phase 1 study using escalating doses of Corlicyte^®^ MSCs applied topically to diabetic foot ulcer twice a week for up to 8 weeks, with at least 4 weeks of follow-up. Non-pregnant adults aged ≥ 18 years with type 1 or type 2 diabetes giving written, informed consent were enrolled in the Wound Clinic at the University of Colorado Hospital in Aurora, Colorado, between November 2019 and December 2021. The study was approved by the WCG Institutional Review Board (IRB) and performed under a United States Food and Drug Administration Investigational New Drug Application.

Corlicyte^®^ is a viable, allogeneic MSC product derived from umbilical cord tissue developed by CellResearch Corporation Pte Ltd. and produced by ClinImmune, an affiliate of the University of Colorado Anschutz Medical Campus. Corlicyte^®^ was produced in accordance with section 501(a)(2)(B) of the Federal Food, Drug, and Cosmetic Act. The cells were derived from umbilical cord tissue following a normal full-term birth by mothers who provided informed consent through an IRB-approved protocol, including a maternal health questionnaire and consent to donate tissue for a commercial product. The MSCs meet the criteria for stem cells according to the International Society for Stem Cell Research (ISSCR) guidelines [14].

The MSCs were manufactured in four stages, as follows: **Stage 1.** Five-centimeter sections of umbilical cords were collected from 100 vaginal or cesarean births in sterile containers containing 30 mL of Plasma-Lyte A (Baxter) supplemented with 50 μg/mL streptomycin sulfate, 50 μg/mL of gentamicin sulfate, and 10 μg/mL ciprofloxacin HCL. Blood samples were collected from the donor mother at the time of delivery and were screened for infectious diseases using the Foundation for the Accreditation of Cellular Therapy (FACT) panel traditionally used for hematopoietic stem cell donors. Tissues used in culture tested negative for microbial contamination. Each 5 cm section of the umbilical cord was washed and cut into forty-eight 2 mm pieces and stored 8 per vial in 1 mL Cryovials containing 60% Plasma-Lyte, 30% of 5% human serum albumin, and 10% DMSO. Cryovials were placed in sterile overwrap bags and frozen to −195 °C at 1 °C/min in a controlled-rate freezer. All tissues were reviewed by the laboratory medical director prior to release for cell expansion. **Stage 2.** Frozen umbilical cord tissues were thawed, washed, and placed in PTT6 culture media supplemented with 2.5% fetal bovine serum, 10 ng/mL epidermal growth factor (Miltenyi), and 5 μg/mL insulin (NovoNordisk) for 2 weeks. Spindle morphology was documented by CytoSmart (Axion). **Stage 3.** The explanted cells from 28 different tissues were screened by flow cytometry and were >98% positive for CD73, CD90, and CD105 and <2% positive for CD34, CD45, and HLA-DR. Two cell lines were selected for expansion on the basis of their ability to produce angiopoietin-1, transforming growth factor-β, vascular endothelial growth factor, and hepatocyte growth factor (R + D Systems multiplex assay) per 10^5^ cells after 48 h in culture media (see Table 1). Two explants were developed into master cell banks by seeding 20 million cells into a Terumo Quantum bioreactor and growing the cells for 5 days, producing > 600 million cells, which were aliquoted at 2 million cells/vial and cryopreserved in 5% DMSO. The master cell banks were tested for HIV1/2, HTLV1/2, EBV, CMV, Parvovirus B19, HCV, HBV, HAV, Herpesvirus 6–8, HPV Type 16 and 18, JC virus, and BK virus by DNA tests and also screened for adventitious viruses on MRC-5, HeLa, 324K, HT1080, A549, HEK293, Vero, LLC-MK2, MDCK, MDBK, CHO K1, NIH3T3, A9, BHK-21, RK13, and SF9 cell lines by Eurofins Lancaster Laboratories (Lancaster, PA). **Stage 4.** Five days prior to use in topical application of a diabetic foot ulcer, a vial of Corlicyte^®^ MSCs was thawed and cultured in a 175 cm^2^ flask. On the day of treatment, the MSCs were harvested and formulated as 1 million freshly cultured cells in HypoThermosol^®^ and delivered to the Wound Clinic in sealed vials at 4 °C.

Subjects qualified for enrollment if the index diabetic foot ulcer was located below the malleolus, extending to the dermis and subcutaneous tissue, without exposed muscle, tendon, or bone, and had a surface area between 0.6 and 12 cm^2^ of duration > 4 weeks. Subjects had a minimum ulcer size of 0.3 cm^2^ at the end of the 2- to 4-week screening period, screening hemoglobin A1c ≤ 130 mmol/mol (14%), and willingness to offload if the index ulcer was plantar. Key exclusion criteria included planned DFU treatment with enzymatic agents or agents that could affect MSC survival, skin disorder around the index ulcer, active infection in the index limb or gangrene/osteomyelitis in either foot, severe peripheral artery disease, or significant titer of antibodies to HLA Class I molecules expressed on Corlicyte^®^ MSCs as measured by Luminex HLA single antigen beads.

All study procedures were performed in the Anschutz Medical Campus Wound Clinic. There were 2 screening visits over a 2- to 4-week period, then twice weekly visits during the treatment phase for up to 8 weeks for debridement when indicated, ulcer measurement and photography using the wound measurement and photograph documentation system eKare^®^, topical application of Corlicyte^®^, and assessment of adverse events (AEs). Corlicyte^®^ was drawn up in a sterile tuberculin syringe, applied topically to the index ulcer, covered with a thin plastic film and sterile gauze, and held in place with self-adherent wrap. Subjects received up to 16 total treatments with Corlicyte^®^. If the ulcer closed before the 8-week treatment phase, the remaining visits were conducted every 2 weeks until the end of the study visit at 12 weeks. Subjects who had open ulcers until the end of the 8-week treatment phase were seen every 2 weeks during the 4-week follow-up phase. Every subject underwent panel reactive antibody measurement every 2 weeks during the trial. Safety laboratory studies and physical exams were assessed at the end of the study visit, which occurred 12 weeks after the first treatment visit. Ulcer measurements were reviewed and confirmed by the Colorado Prevention Center (CPC) Wound Core Lab, which was independent of the study site staff.

**Figure 1:**
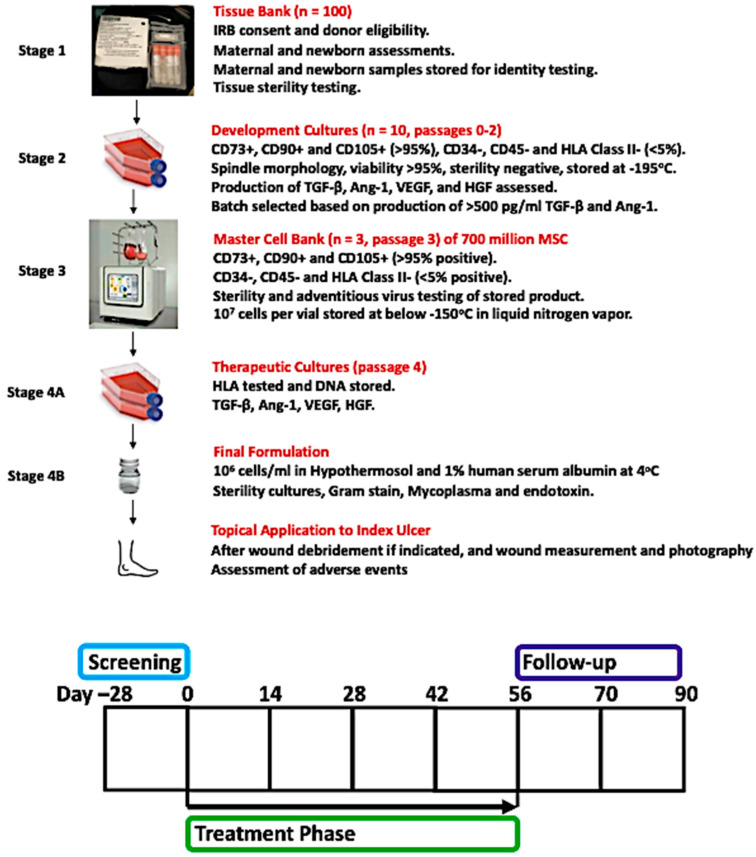
Diagram of manufacturing steps and subject participation. Manufacturing steps are further described in Methods. Subjects were seen twice weekly in the Wound Clinic during the treatment phase and underwent wound debridement if indicated, wound measurement and photography, and Corlicyte^®^ was applied topically to the index ulcer. If the ulcer had closed, subjects continued to be seen at landmark visits every 2 weeks, and all subjects were seen for follow-up through Day 90 for study procedures including assessment of adverse events.

**Figure 2:**
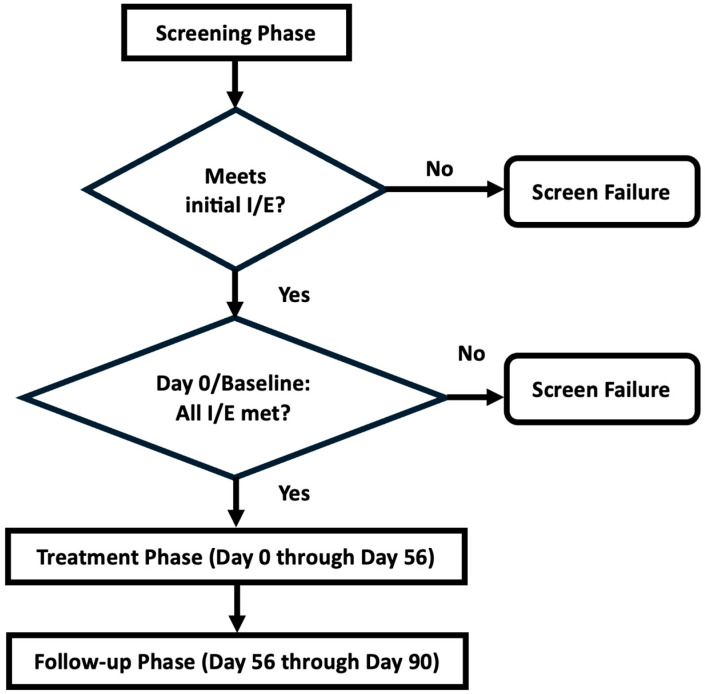
Study phases. After obtaining informed consent, subjects underwent screening. If they still met inclusion and exclusion criteria at the baseline, they entered the treatment phase. The follow-up phase involved visits every 2 weeks for assessment of the ulcer site, adverse events, and panel reactive antibody measurement. Final safety laboratory testing was performed at the last visit. I/E: inclusion/exclusion criteria.

The goal was to have 5 subjects receive Corlicyte^®^ in each of the three dosing cohorts (Cohort A: 100,000 cells/mm^3^, Cohort B: 300,000 cells/mm^3^, Cohort C: 500,000 cells/mm^3^ ulcer surface area). The Dose Escalation Committee (DEC) met to assess the safety and tolerability of Cohort A after 5 subjects had completed the Day 28 visit before Cohort B could begin treatment. Criteria for discontinuation of treatment with IP included (1) Common Terminology Criteria for Adverse Events (CTCAE) Grade three (3) or greater allergic reaction at the index ulcer site during the Treatment Phase, or (2) significant worsening of the index ulcer during the treatment phase. Please see Figure 1 for a diagram depicting the manufacturing steps and timeline of subject participation and Figure 2 for the study phases.

The primary endpoint was the number and percent of subjects in each dosing cohort and overall with a serious adverse reaction to Corlicyte^®^. Key secondary endpoints included the number and percent of subjects who developed a high titer of antibodies to human leukocyte antigen (HLA) Class I molecules expressed on Corlicyte^®^ and the number and percent of subjects with an increase in ulcer size by the end of the treatment phase as reviewed by the CPC Wound Core Laboratory. Descriptive statistics were used to summarize baseline characteristics of subjects in each cohort and overall. Adverse events were coded using version 21.0 or higher of the Medical Dictionary for Regulatory Activities (MedDRA). Incidence and types of AEs including allergic reactions and development of high titer antibodies to HLA Class I molecules expressed on Corlicyte^®^ MSCs were summarized for each cohort and overall.

## 3. Results

### 3.1. Enrollment of Cohorts

Sixteen subjects consented and 7 failed screening, with 9 subjects undergoing treatment and completing the trial: 5 subjects in Cohort A and 4 subjects in Cohort B. The DEC met in October 2021 and allowed the trial to proceed. A third dosing cohort was planned but not performed due to slow enrollment after the onset of the COVID-19 pandemic. Baseline demographics and characteristics are given in Table 2 and Table 3.

### 3.2. Adverse Events

Twelve adverse events including one serious adverse event (pneumonia) were reported in 6 subjects, all deemed unlikely related to the investigational product, and not resulting in dose change, interruption, or discontinuation of treatment. Eight of the adverse events were mild (upper respiratory tract infection, urinary tract infection, fungal infection, road traffic accident, skin irritation, skin ulcer, cellulitis, wound, pain in extremity), one was moderate (pyelonephritis), and two were severe (pneumonia, fall), including the SAE resulting in hospitalization (pneumonia). Adverse events are detailed in Table 4.

### 3.3. Endpoints and Safety Laboratory Results

No subject developed a serious adverse reaction to Corlicyte^®^, the primary endpoint, and no subject developed anti-HLA antibodies at any time during the trial. The number of subjects with an increase in ulcer size by the end of the treatment phase as reviewed by the Wound Core Laboratory was zero. Safety laboratory testing was unremarkable (Table 3).

### 3.4. Ulcer Closure and Ulcer Size Reduction

**Figure 3:**
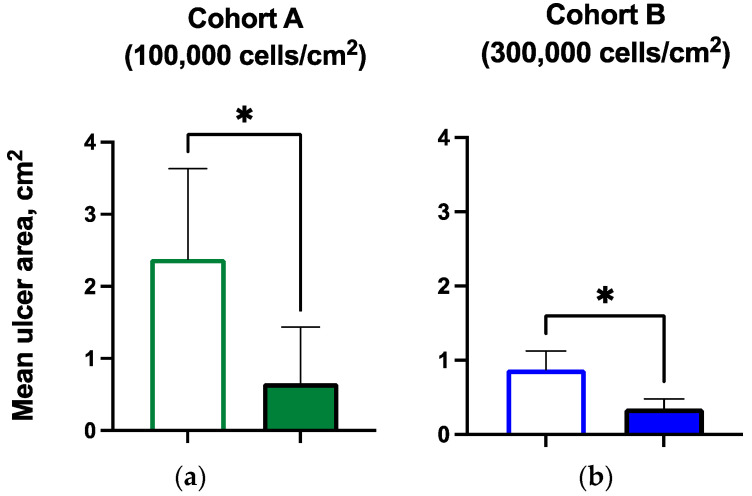

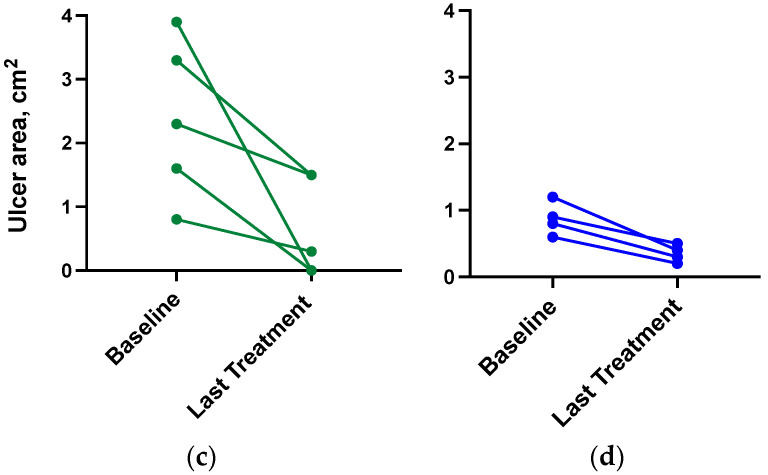
Ulcer area at baseline and last treatment visit by cohort, aggregate, and by subject (* *p* < 0.05): (**a**) mean ulcer area for subjects in Cohort A at baseline and last treatment visit; (**b**) mean ulcer area for subjects in Cohort B at baseline and last treatment visit; (**c**) Cohort A individual subject ulcer area at baseline and last treatment; (**d**) Cohort B individual subject ulcer area at baseline and last treatment.

Three of five subjects in the lower dose cohort experienced ulcer closure by Day 70 of follow-up. All of the subjects experienced a reduction in ulcer area or ulcer closure by the last visit. Of the six subjects with open ulcers at the last treatment visit, the mean ulcer size was reduced significantly, by 54% and 67%, respectively, in the two dosing cohorts (Table 5, Figure 3).

### 3.5. Hemoglobin A1c

The mean A1c decreased slightly in Cohort A, from 10.1% at baseline to 9.6% at the end of the study. The numerical decrease in A1c was greater in Cohort B, from a mean of 10.7% at baseline to 8.7% at the end of the study (Table 5).

## 4. Discussion

This phase 1 trial demonstrates that topical administration of Corlicyte^®^ MSC, a novel biologic therapy consisting of allogeneic umbilical cord lining mesenchymal stem cells, did not induce the development of anti-HLA antibodies and did not cause adverse events. There was a significant decrease in ulcer area from baseline to last treatment in both dosing cohorts. MSCs have been demonstrated to contribute to tissue repair and maintenance throughout the body [13]. The potential mechanism has been investigated in an animal model using human MSCs applied topically in a wound model in db/db mice with impaired wound healing [13]. In this model, an increased number of host MSCs were recruited to the wound bed despite the rapid loss of human MSCs, which suggested a paracrine effect of the grafted MSCs, with mobilization of a host response that contributed to wound repair [13]. Promotion of ulcer repair by stem cells without requiring injection either intravenously, subcutaneously, or intramuscularly would be anticipated to minimize the likelihood that potentially adverse anti-HLA antibodies would be produced, which is consistent with what was demonstrated in this trial. MSC cryopreservation and preservation of therapeutic function are areas of active investigation [15], and several steps were taken in this trial to preserve MSC function including controlled-rate freezing with the use of low concentration of DMSO, and removal by washing prior to culture recovery, and the 5-day time frame used for culture recovery prior to topical application. A search in PubMed for clinical trials of MSC therapy for DFU yielded mostly bone marrow- or adipose-derived MSCs administered via injection or infusion, but none that were derived from umbilical cord tissue and topically administered, yet these are characteristics anticipated to increase the scalability and ease of administration of this therapy. Strengths of this study include the level of rigor for all steps of manufacturing and conduct of the clinical trial to meet regulatory agency requirements, and the use of a wound core laboratory to verify ulcer measurements. The main limitations of this study are the small sample size and lack of a control group; but as a phase 1 first-in-human study, an initial demonstration of safety and tolerability was necessary prior to exposure in a larger clinical trial. In conclusion, this trial supports the assertion that Corlicyte^®^ warrants further study as a potential therapy for the healing of chronic diabetic foot ulcers.

## Figures and Tables

**Table 1:** Quantification of cytokine production for cell lines selected for expansion. Ang-1: angiopoietin-1; HGF: hepatocyte growth factor; TGF: transforming growth factor; VEGF: vascular endothelial growth factor.

Mean (Range), pg/mL	Cohort A, *n* = 5	Cohort B, *n* = 4
TGF-β	1198 (549–2610)	1793 (749–6237)
Ang-1	1128 (900–1357)	677 (84–1733)
VEGF	232 (211–254)	790 (101–2280)
HGF	357 (276–438)	764 (54–2395)

**Table 2:** Subject characteristics at baseline: overall and by cohort. DPP4i: dipeptidylpeptidase inhibitor-4; GLP1RA: glucagon-like-peptide-1 receptor agonist; SGLT2i: sodium–glucose cotransporter-2 inhibitor.

	Overall, *n* = 9	Cohort A, *n* = 5	Cohort B, *n* = 4
Age (yr): mean (range)	55 (44–70)	56 (45–70)	54 (44–60)
Female/male (number)	3/6	2/3	1/3
White/Black (number)	8/1	4/1	4/0
Type 2 diabetes/Type 1 diabetes	8/1	4/1	4/0
Diabetes duration (yr): mean (range)	24 (5–49)	33 (21–49)	12 (5–16)
Ulcer chronicity (months): mean (range)	7.3 (3–13)	7.7 (3–15)	6.8 (1.7–13.5)
Diabetes medication use: *n* (%)	9 (100%)	5 (100%)	4 (100%)
Insulin	8 (89%)	5 (100%)	3 (75%)
Non-insulin	4 (44%)	2 (40%)	2 (50%)
Metformin	3 (33%)	1 (20%)	2 (50%)
GLP1RA	0	0	0
DPP4i	4 (44%)	2 (40%)	2 (50%)
SGLT2i	0	0	0
Sulfonylurea	2 (22%)	1 (20%)	1 (25%)
Thiazolidinedione	1 (11%)	1 (20%)	0

**Table 3:** Hemoglobin A1c and selected safety labs at baseline and last visit. WBC: white blood cell.

*n* = 9	Baseline	Last Visit
Hemoglobin A1c: mean (range)		
mmol/mol	90.2 (58.5–107.7)	76.0 (44.3–113.1)
%	10.4 (7.5–12)	9.1 (6.2–12.5)
WBC (×10^3^/mm^3^): mean (range)	8.8 (4–13.7)	9.2 (6.4–12.9)
Creatinine (mg/dL): mean (range)	0.9 (0.5–1.4)	0.9 (0.6–1.5)

**Table 4:** Adverse events by cohort and overall. SAE: serious adverse event.

Adverse Event (AE)	Cohort A Corlicyte^®^ 100,000 cells/cm^2^ *n* = 5	Cohort B Corlicyte^®^ 300,000 cells/cm^2^ *n* = 4	Total Overall *n* = 9
Number of AEs	11	1	12
Number (%) of patients with at least one AE	5 (100.00%)	1 (25.00%)	6 (66.67%)
Number (%) of patients with at least one AE related to study treatment	0	0	0
Number (%) of patients with at least one AE leading to study drug withdrawal	0	0	0
Number (%) of patients with adverse reactions to study drug	0	0	0
Number (%) of patients with at least one SAE	1 (20.00%)	0	1 (11.11%)
Number (%) of patients with at least one SAE related to study treatment	0	0	0
Number of AEs by severity			
Grade 1 Mild	8	1	9
Grade 2 Moderate	1	0	1
Grade 3 Severe	2	0	2
Grade 4 Life-threatening	0	0	0
Grade 5 Death	0	0	0

**Table 5:** Ulcer size and hemoglobin A1c by cohort. Ulcer size at baseline and last treatment visit; hemoglobin A1c at baseline and Day 90 visit.

	Cohort A *n* = 5		Cohort B *n* = 4	
	Baseline	Day 90 Visit	Baseline	Day 90 Visit
Hemoglobin A1c (mean)				
mmol/mol	86.9	81.4	93.4	71.6
%	10.1	9.6	10.7	8.7
* *p* < 0.05	**Baseline**	**Last Treatment Visit**	**Baseline**	**Last Treatment Visit**
Ulcer size (mean, cm^2^)	2.38	0.66 *	0.88	0.35 *

## Data Availability

The data that support the findings of this study are available from CellResearch Corp.
